# IL-2 Expression in Activated Human Memory FOXP3^+^ Cells Critically Depends on the Cellular Levels of FOXP3 as Well as of Four Transcription Factors of  T Cell Activation

**DOI:** 10.3389/fimmu.2012.00264

**Published:** 2012-08-27

**Authors:** Hanna Bendfeldt, Manuela Benary, Tobias Scheel, Kerstin Steinbrink, Andreas Radbruch, Hanspeter Herzel, Ria Baumgrass

**Affiliations:** ^1^German Rheumatism Research Centre Berlin, a Leibniz InstituteBerlin, Germany; ^2^Institute for Theoretical Biology, Humboldt University of BerlinBerlin, Germany; ^3^Department of Dermatology, Johannes Gutenberg University of MainzMainz, Germany; ^4^CharitéBerlin, Germany

**Keywords:** cytokine expression, transcription factors, T cell activation, IL-2 expression, lymphocyte, flow cytometry, human Treg cells, memory Th cells

## Abstract

The human CD4^+^FOXP3^+^ T cell population is heterogeneous and consists of various subpopulations which remain poorly defined. Anergy and suppression are two main functional characteristics of FOXP3^+^Treg cells. We used the anergic behavior of FOXP3^+^Treg cells for a better discrimination and characterization of such subpopulations. We compared IL-2-expressing with IL-2-non-expressing cells within the memory FOXP3^+^ T cell population. In contrast to IL-2-non-expressing FOXP3^+^ cells, IL-2-expressing FOXP3^+^ cells exhibit intermediate characteristics of Treg and Th cells concerning the Treg cell markers CD25, GITR, and Helios. Besides lower levels of FOXP3, they also have higher levels of the transcription factors NFATc2, c-Fos, NF-κBp65, and c-Jun. An approach combining flow cytometric measurements with statistical interpretation for quantitative transcription factor analysis suggests that the physiological expression levels not only of FOXP3 but also of NFATc2, c-Jun, c-Fos, and NF-κBp65 are limiting for the decision whether IL-2 is expressed or not in activated peripheral human memory FOXP3^+^ cells. These findings demonstrate that concomitant high levels of NFATc2, c-Jun, c-Fos, and NF-κBp65 lead in addition to potential IL-2 expression in those FOXP3^+^ cells with low levels of FOXP3. We hypothesize that not only the level of FOXP3 expression but also the amounts of the four transcription factors studied represent determining factors for the anergic phenotype of FOXP3^+^ Treg cells.

## Introduction

FOXP3-expressing Treg cells are essential for the maintenance of immunological self-tolerance and immune homeostasis. CD4^+^FOXP3^+^ Treg cells are able to suppress the activation, proliferation, and effector functions of many different immune cells such as Th cells, cytotoxic T cells, NK cells, and APCs. These Treg cells play a central role in preventing autoimmune diseases and allergies as demonstrated in human diseases and animal models. Compared to murine FOXP3^+^ T cells, human FOXP3^+^ T cells are more heterogeneous concerning their phenotypical and functional properties resulting in various FOXP3^+^ subpopulations (reviewed in Sakaguchi et al., 2010; Miyara and Sakaguchi, 2011). Furthermore, not all peripheral human CD4^+^FOXP3^+^ cells are suppressive (Baecher-Allan et al., 2001). This high complexity of human FOXP3^+^ T cells causes some confusion in the field. Therefore, many efforts were undertaken to discriminate between suppressive and non-suppressive subpopulations among the *ex vivo* isolated CD4^+^FOXP3^+^ human T cells. Most promising so far is the division of FOXP3^+^ cells into three subpopulations based on FOXP3 and CD45RA or CD45RO expression: (i) naïve Treg cells (CD45RA^+^FOXP3^low^); (ii) memory effector Treg cells (CD45RA^−^FOXP3^high^); and (iii) memory non-suppressive Th cells (CD45RA^−^FOXP3^low^; Miyara et al., 2009).

A detailed knowledge of human FOXP3^+^ cell subsets is essential for a better understanding of the regulation of human FOXP3 expression, the study of abnormalities among the FOXP3^+^ subpopulations in autoimmune diseases and allergies, and the selection and purification of the most promising subpopulation(s) of Treg cells for *in vitro* expansion and adoptive transfer (Fujii et al., 2011; Miyara and Sakaguchi, 2011).

In this study we aimed at a better characterization of human memory FOXP3-expressing T cell subpopulations (CD4^+^CD45RO^+^FOXP3^+^) concerning anergy defined by the inability to produce proinflammatory cytokines upon TCR/CD28 stimulation. Anergy and suppression are two main functional characteristics of FOXP3^+^ Treg cells (Gavin et al., 2007; Wan and Flavell, 2007; Williams and Rudensky, 2007). Therefore, we compared IL-2-expressing and IL-2-non-expressing FOXP3^+^ memory T cells using Treg cell markers and the expression levels of five transcription factors. We used measurement of cytokine expression, in particular IL-2, by T cells as a substitute for suppression assays, because it was hard to get enough pure cells after sorting of the subpopulations. Performing a combination of flow cytometric measurements with statistical analysis (Bendfeldt et al., 2012) we studied the impact of low, medium low, medium high, and high levels of the transcription factors FOXP3, NFATc2, c-Jun, c-Fos, or NF-κBp65 on the decision whether IL-2 is expressed or not in activated memory FOXP3^+^ cells. Results of our data analysis suggest that low levels of FOXP3 expression in combination with high levels of NFATc2, c-Jun, c-Fos, or NF-κBp65 are critically required for IL-2 expression of FOXP3^+^ T cells.

## Materials and Methods

### Human T cell isolation, stimulation, and staining

Peripheral blood mononuclear cells from healthy volunteers were prepared using Ficoll PAQUE gradients from leukocyte concentrates obtained from the blood bank of the red cross. Positively selected CD4^+^ cells were depleted of CD45RA^+^ Th cells to a purity of >97% CD4^+^CD45RO^+^ memory Th cells (MACS Separation Reagents; Miltenyi Biotech). After resting over night cells were cultured in RPMI supplemented with 10% fetal calf serum and stimulated either with 10 ng/ml PMA (Sigma) and 1 μg/ml Ionomycin (Sigma) or with anti-CD3/CD28 antibody-coupled beads (25 μl per 1 × 106 cells; Dynal Beads, Invitrogen). Brefeldin A (Sigma) was added 30 min after stimulation to inhibit IL-2 secretion and to trap the expressed IL-2 within the cells. For cytometric analysis cells were stained for surface molecules with anti-CD69-APC (Miltenyi) and anti-CD25-PE-Cy7 antibodies (BD). For intracellular staining cells were fixed and permeabilized using the FOXP3 staining buffer set (eBioscience) and stained with antibodies against FOXP3-FITC (eBioscience), IL-2-APC (BD), IL-4-PE (BD), IL-17-PE (eBioscience), IFN-γ-Pacific orange (own in-house antibody), NFATc2-FITC (own antibody, BD), c-Fos-A488 (Santa Cruz Biotechnology), phospho-c-Jun-A488 (Santa Cruz Biotechnology), NF-κBp65-A488 (Santa Cruz Biotechnology), GITR-PE (Miltenyi), or Helios-PE (Biolegend). Subsequently, cells were analyzed using a LSR II Fortessa (BD). The MEK1/2 inhibitor U0126 (Biomol GmbH) was pre-incubated with Th cells for 20 min before stimulation and was used in concentrations of 0.01–250 μM.

### Isolation of CD25^−^CD127^+^ cells by FACS

Sorted CD4^+^CD45RO^+^ memory Th cells were stained with antibodies against CD25-PE-Cy7 (BD) and CD127-PE (Beckman Coulter) and sorted using a FACS Aria (BD).

### Data analysis and mathematical modeling

The free software R was used for data analysis. Sorted memory Th cells (CD4^+^CD45RO^+^) were gated for FOXP3 positive cells. Quantification was performed by transforming the fluorescence intensities of the cells into a linear scale using the asinh function. The range of transcription factor expression levels was divided either into four bins with equal cell numbers or into six bins with equidistant expression levels resulting in uneven cell number distribution. For each bin the number of IL-2-producing cells was calculated. Changes in mean expression levels were tested for significance using a paired Student *t*-test.

Mathematical modeling was done using Matlab. For each data set the parameters of the heuristic model were estimated by minimizing the distance from the simulated data to the experimental data (least square fitting). To compare models with different number of parameters we used Akaike’s information criterion with correction for finite sample sizes (AICc), which takes into account the distance between model and data as well as considering the complexity of the model based on the number of parameters.

## Results

### Up to one-fifth of peripheral human memory FOXP3^+^ cells are able to express IL-2

**Figure 1:**
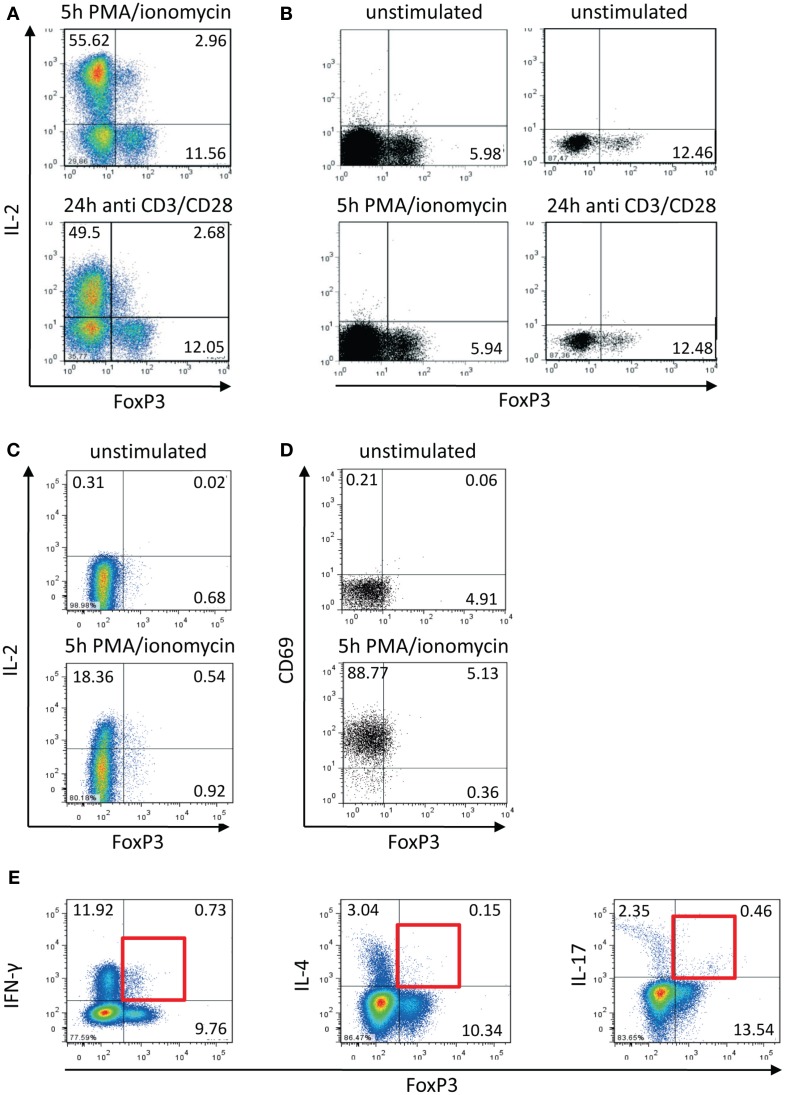
**Activation-induced expression of IL-2 within the FOXP3^+^ memory T cell population**. Human CD4^+^CD45RO^+^ T cells were isolated using magnetic cell-sorting. **(A,B)** Cells were stimulated with PMA/ionomycin and antibodies against CD3/CD28 for 5 and 24 h, respectively, and subsequently stained with fluorophore-coupled antibodies and analyzed by flow cytometry. **(C)** CD4^+^CD45RO^+^ T cells were stained with fluorophore-coupled antibodies against CD25 and CD127. Subsequently CD25^−^CD127^+^ cells were sorted by FACS, stimulated with PMA/ionomycin for 5 h, and subsequently fixed, stained, and analyzed by flow cytometry. **(D,E)** PMA/ionomycin-stimulated CD4^+^CD45RO^+^ T cells (5 h) were fixed, stained with fluorophore-coupled antibodies, and analyzed by flow cytometry. Data are representative out of two **(C–E)** or more than three **(A,B)** independent experiments.

Comparing transcription factor levels and cytokine expression within the memory CD4^+^ T cell population of healthy donors (Bendfeldt et al., 2012) we discovered that there are always 8–20% IL-2-expressing FOXP3^+^ Th cells, in spite of the fact that FOXP3^+^ cells are widely considered to be incapable of producing IL-2 and other effector cytokines (Hori et al., 2003). The IL-2-expressing FOXP3^+^ cells were induced by stimulation with PMA/ionomycin as well as with anti-CD3/CD28 antibodies (Figure 1A). On the one hand, we confirmed that this population did not emerge from stimulation-dependent up-regulation of FOXP3 in FOXP3^−^ memory Th cells. First, the frequency of FOXP3^+^ T cells did not change within the studied time frame of 5 h after stimulation (Figure 1B). Second, sorted memory Th cells (CD25^−^CD127^+^) that are depleted of Treg cells (CD25^high^CD127^low^), did not up-regulate FOXP3 expression after stimulation with PMA/ionomycin for 5 h (Figure 1C). On the other hand, staining of the activation marker CD69 demonstrated that all FOXP3^+^ T cells were activated equally under the stimulation conditions employed (Figure 1D).

We already knew that approximately 35% (+5%) of IL-2-expressing activated memory Th cells are co-expressing IFN-γ (Podtschaske et al., 2007 and data not shown). Here, we observed similar frequencies of IFN-γ^+^ cells (approximately 30%) within the IL-2^+^FOXP3^+^ cell population (data not shown). Among the whole entity of FOXP3^+^ cells there were only low frequencies of cells expressing IFN-γ (between 5 and 10%) and IL-17 as well as IL-4 (both under 5%) after stimulation (Figure 1E).

### IL-2-expressing FOXP3^+^ cells express a lower level of phenotypic markers of Treg cells

**Figure 2:**
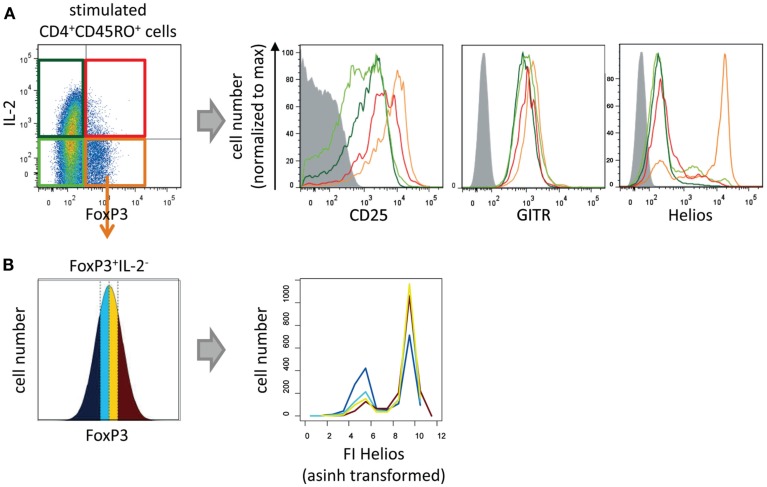
**Phenotypical characterization of IL-2-expressing FOXP3^+^ cells**. Sorted human CD4^+^CD45RO^+^ T cells were stimulated with PMA/ionomycin for 5 h and subsequently fixed, stained with fluorophore-coupled antibodies, and analyzed by flow cytometry. **(A)** Histograms show overlays of CD25, GITR, or Helios expression of gated IL-2^−^FOXP3^−^ (light green), IL-2^+^FOXP3^−^ (dark green), IL-2^−^FOXP3^+^ (orange), and IL-2^+^FOXP3^+^ cells (red). **(B)** Within the gated FOXP3^+^ IL2^−^ cell population, the range of FOXP3 expression level per cell was divided into quartiles (with the same cell number). The effect of the FOXP3 expression levels on Helios expression is shown in histogram overlays. Results of one representative experiment are shown (**A,B**: *n* = 2).

To discover phenotypic differences between IL-2-non-expressing and IL-2-expressing CD4^+^ FOXP3^+^ T cells we analyzed the levels and frequencies of the Treg cell markers CD25, GITR, and Helios in both subpopulations and compared them both with memory Th cells (Figure 2). As expected IL-2^−^ FOXP3^+^ cells express CD25, GITR, and Helios (orange line), whereas all memory FOXP3^−^ T cells have a lower level of CD25 and GITR and were negative for Helios (light and dark green lines). IL-2^+^ FOXP3^+^ showed an intermediate expression level for CD25 and GITR and were negative for Helios expression (red line) indicating a cellular phenotype between Treg and memory Th cells.

Specifically, to study whether Helios expression is dependent on the level of FOXP3 per cell we divided the FOXP3 fluorescence intensity (FI) of the IL-2^−^FOXP3^+^ subpopulation into quartiles. Each quartile contained the same number of cells (Figure 2B, left). Subsequently, the Helios expression in each quartile was plotted into a histogram (Figure 2B, right). The level of FOXP3 per cell showed a clear correlation with the number of Helios-expressing FOXP3^+^ cells.

### IL-2-expressing FOXP3^+^ cells express more NFATc2 and AP-1 but less FOXP3 than IL-2-non-expressing FOXP3^+^ cells

Recently, we discovered that sorted human memory Treg cells (CD25^high^CD127^low^) cells express lower levels of NFATc2, c-Fos, NF-κBp65, and c-Jun than Treg-depleted memory Th cells (CD25^low^CD127^high^) by western blotting (Bendfeldt et al., 2012). These differences motivated us to examine whether the low transcription factor expression observed in Treg cells is also a general feature of IL-2-non-expressing, as compared with IL-2-expressing, FOXP3^+^ cells.

**Figure 3:**
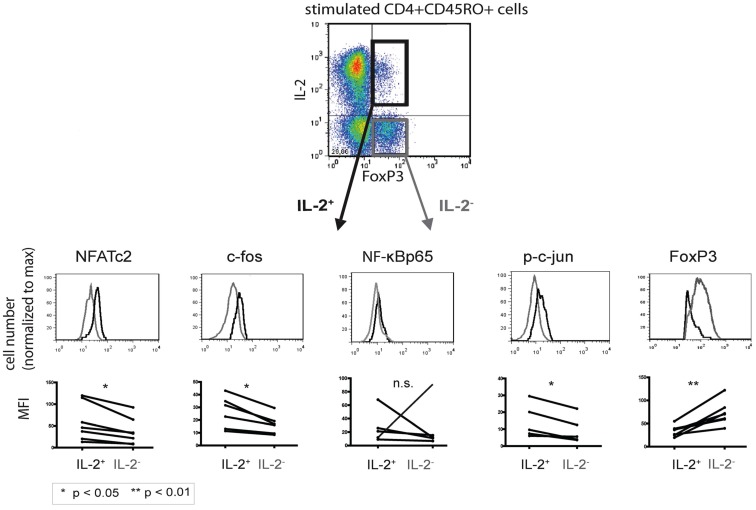
**Comparison of the transcription factor expression levels between IL-2-expressing and -non-expressing FOXP3^+^ cells**. PMA/ionomycin-stimulated human CD4^+^CD45RO^+^ T cells were fixed, stained with fluorophore-coupled antibodies, and analyzed by flow cytometry. IL-2^+^ (black) and IL-2^−^ (gray) FOXP3^+^ T cells were gated and analyzed for the respective transcription factors. Displayed are histogram overlays of the transcription factor expression in the two subpopulations of one representative donor (*n* = 5 or 6). Differences in the mean fluorescence intensity (MFI) of the transcription factors between IL-2-expressing and -non-expressing FOXP3^+^ Th cells in five or six different donors are shown as graphs. Significance was determined using a paired student *t*-test.

Established co-stainings for IL-2 and the respective transcription factors (Bendfeldt et al., 2012) enabled us to directly compare quantitative differences in the expression levels of NFATc2, c-Fos, NF-κBp65, p-c Jun, and c-Fos in IL-2^+^ and IL-2^−^ FOXP3^+^ cells (Figure 3, upper part). Flow cytometric analysis of PMA/ionomycin-stimulated memory Th cells from five to seven different donors showed a higher expression of NFATc2 (*p* = 0.0105), c-Fos (*p* = 0.0076), and p-c-Jun (*p* = 0.0197) in IL-2-expressing than in IL-2-non-expressing FOXP3^+^ cells (Figure 3, lower part). No significant differences were observed in the expression of NF-κBp65 between IL-2^+^ and IL-2^−^ FOXP3^+^ cells (*p* = 0.9988). In order to stain c-Jun, we had to use a phospho-specific antibody because co-staining of c-Jun together with IL-2 and FOXP3 was not possible so far. Interestingly, the expression of FOXP3 was much lower (*p* = 0.0030) in IL-2^+^ than in IL-2^−^ FOXP3^+^ cells (*n* = 6; Figure 3). CD3/CD28-stimulated memory Th cells showed similar results (data not shown).

Several reports revealed defects in the proximal TCR-signaling cascade in FOXP3^+^ Th cells (Hickman et al., 2006; Carson and Ziegler, 2007). Recently, we could rule out that these defects have an impact on the activation of the main transcription factors at strong stimulation, and therefore that they play a decisive role for IL-2 expression in FOXP3^+^ T cells under these conditions (Bendfeldt et al., 2012). Using co-staining and flow cytometric measurement of nuclei we demonstrated that the activation of NFATc2, c-Fos, and NF-κBp65 is unaffected after stimulation of FOXP3^+^ cells, both with PMA/ionomycin and CD3/CD28 stimulation. Therefore, the repression of IL-2 expression in FOXP3^+^ cells is not due to impaired activation of NFATc2, c-Fos, c-Jun, and NF-κBp65.

### Low levels of FOXP3 and high levels of NFATc2, c-Jun, c-Fos, and NF-κBp65 are important for IL-2 decision making in FOXP3^+^ cells

Testing the hypothesis that physiological levels of FOXP3 are limiting for IL-2 expression in primary Treg cells usually requires over-expression or knock-down of FOXP3. However, such manipulations of primary Th cells often lead to a different *status quo* not only of one transcription factor but also of the complete transcription factor network compared to unmodified *ex vivo* cells.

**Figure 4:**
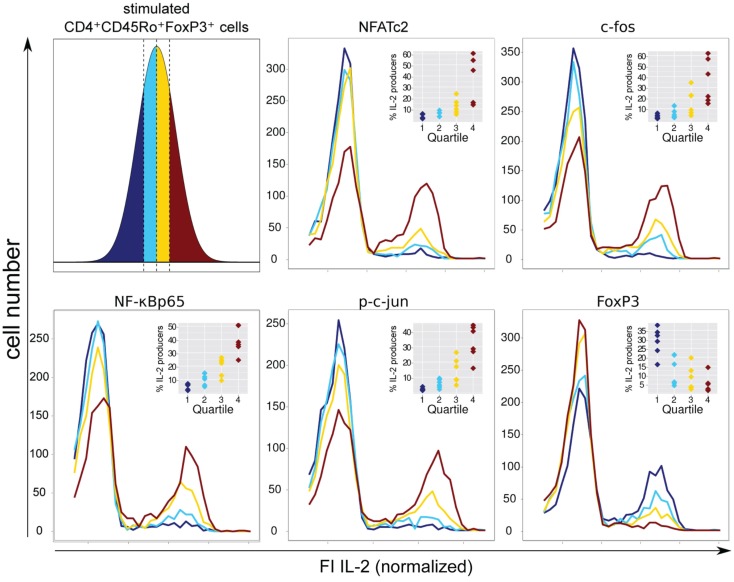
**Negative correlation of FOXP3 and positive correlation of NFATc2, c-Fos, NF-κBp65, and c-Jun expression level with IL-2 expression within the FOXP3^+^ population**. Sorted human CD4^+^CD45RO^+^ T cells were stimulated with PMA/ionomycin for 5 h, fixed and stained with fluorophore-coupled antibodies against FOXP3, intracellular IL-2 and in parallel with one of the transcription factors NFATc2, c-Fos, NF-κBp65, or p-c-Jun. Data from the gated FOXP3^+^ cell population were transformed into a linear scale using the asinh function. Subsequently, the range of transcription factor expression level was divided into quartiles (with the same cell number) according to the level of expression of each transcription factor at single cell level (upper left). The effect of different transcription factor expression levels on IL-2 expression is shown in histogram overlays of one experiment. The inserts show the frequencies of IL-2-expressing FOXP3^+^ cells within the quartiles from six different donors.

Therefore, we decided to circumvent these difficulties, using a novel approach combining flow cytometry data analysis with statistical evaluation of single cell data. Within the FOXP3^+^ cell population of sorted and activated memory Th cells (CD4^+^CD45RO^+^) of six healthy donors, we correlated the physiological level of five transcription factors with IL-2 expression per single cell. Specifically, for each transcription factor we divided the FI, as a measure of the protein level per cell, into quartiles. Each quartile contained the same number of cells (Figure 4, upper left). Subsequently, the IL-2 expression in each quartile was plotted into a histogram (Figure 4). The level of the four transcription factors NFATc2, c-Fos, c-Jun, and NF-κBp65 showed an impressive correlation with the number of IL-2-expressing FOXP3^+^ cells. This is in contrast to the observed data concerning memory FOXP3^−^ cells where only c-Fos and to a lower amount NFATc2 but not c-Jun and NF-κBp65 limit IL-2 expression (Bendfeldt et al., 2012).

FOXP3 itself exhibits a clear negative correlation with the number of IL-2-producing FOXP3^+^ cells. As expected, quartiles with higher levels of FOXP3 (yellow and ruby color) have lower numbers of IL-2-expressing cells. In contrast, quartiles with very low (dark blue color) and relatively low (blue color) levels of FOXP3 per cell exhibit the highest numbers of IL-2 -expressing cells, namely 28.7 ± 7.9 and 12.4 ± 8.0%, respectively (Figure 4, insets).

**Figure 5:**
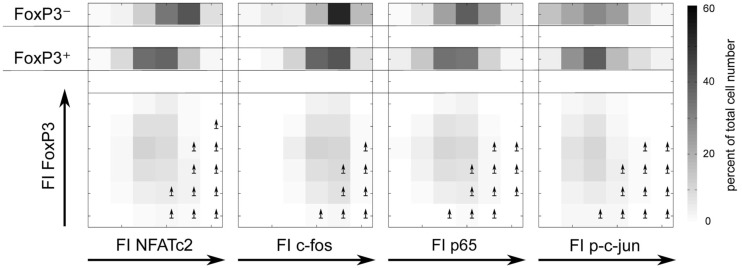
**The ratios of FOXP3 and NFATc2, c-Fos, NF-κBp65, or c-Jun expression level are important for a high probability of IL-2 expression**. Sorting, stimulation, and staining of the cells were identical as described in Figure 4. The range of the fluorescence intensity (FI) of each transcription factor was divided into six bins. The cell numbers are depicted in a gray scale. Arrows label the bins that exhibit more than 30% of IL-2-expressing cells.

To verify that the level of the studied transcription factors NFATc2, c-Jun, c-Fos, and NF-κBp65 correlates with IL-2 expression in FOXP3^+^ cells, we divided the FI of each transcription factor into six bins and depicted the cell numbers as well as the frequencies of IL-2 expression in each bin (Figure 5, lower part). Obviously, the ratio of FOXP3 and the respective transcription factor is important for a high probability of IL-2 expression (arrows are depicted in the bins with more than 30% IL-2-expressing cells). Comparison of cell numbers of memory FOXP3^+^ with FOXP3^−^ cells (Figure 5, upper part) revealed that the whole population of FOXP3^+^ cells shifted to a lower level of transcription factors, clearly visible for NFATc2, c-Fos, and NF-κBp65. Higher cell numbers are in bins (darker bins) with a lower level of these transcription factors. However, with this kind of studies we cannot answer the question whether the reduced expression of these transcription factors is a direct or indirect effect of FOXP3.

#### A minimal heuristic model estimates the balance of transcription factors regulating IL-2 production in FOXP3^+^ cells

To better understand the underlying mechanisms of IL-2 expression in human memory FOXP3^+^ cells we combined our experimental data with a minimal model of transcriptional regulation.

Most models assume that the rate of transcription of a certain gene is a function of the concentrations of the active transcription factors. The cytokine IL-2, however, is expressed in an all-or-none fashion (Podtschaske et al., 2007; Smith and Popmihajlov, 2008). Therefore, the level of IL-2 per cell remains constant after successful activation. Consequently, we modeled the number of IL-2-expressing cells instead of IL-2 expression level as a function of transcription factor concentration.

To quantify the experimental findings, we used a heuristic model with one activating variable *x*_1_ and one inhibiting variable *x*_2_

(1) $$y=\frac{f\cdot x_{\text{1}}+b}{\text{1}+c_{\text{1}}\cdot x_{\text{1}}+c_{\text{2}}\cdot x_{\text{2}}},$$

where *b* and *f* correspond to the basal and the maximal response level, respectively. The parameters *c*_1_ and *c*_2_ reflect the effects of the activating and the inhibiting transcription factors. The activating variable *x*_1_ represents the concentrations of the transcription factors c-Fos, NFATc2, NF-κBp65, and p-c-Jun and the inhibiting variable *x*_2_ corresponds to the concentration of FOXP3. The dependent variable *y* reflects the fraction of cells that are able to express IL-2 and therefore should be in the range between 0 and 1. For simplicity, the parameters *b* (=0) and *f* (=1) were kept constant, whereas *c*_1_ and *c*_2_ were estimated by minimizing the distance from the simulated data to the experimental data (least square fitting) for each combination of transcription factors available. The explained variance *R*^2^ is a measure for the goodness of a fit and describes the proportion of variability (or variance) of a data set, which is accounted by a model. This simple model successfully explains up to 55% of the variance of the data within one donor, leaving the remaining variance open to experimental and technical noise. Relaxing the model constraints to allow _f_ to be fitted as well, leads to similar explained variance, but the Akaike’s value as a measure of goodness of fit favors the two-parameter model.

**Figure 6:**
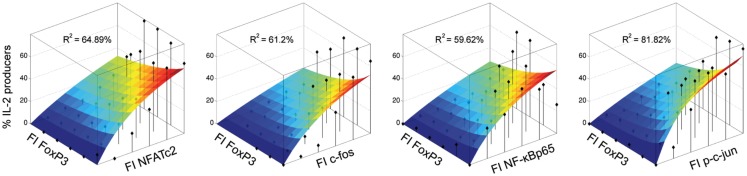
**Modeling confirms that NFATc2, c-Fos, c-Jun, and NF-κBp65 have an activating effect on the frequency of IL-2-expressing cells within the FOXP3^+^ population**. Sorted human Treg cells stimulated with PMA/ionomycin for 5 h were analyzed in parallel for IL-2, FOXP3, and one activating transcription factor per cell by flow cytometry. The normalized expression levels of the transcription factors were divided into 2D-bins. The fraction of IL-2-expressing cells was calculated for each bin, which contained at least five cells (black diamonds). A heuristic model including an activating and an inhibiting term was fitted to the data. The landscape of the model for each data set (each TF from one donor) is superimposed on the data and the *R*^2^ value is shown.

The modeling confirms that the studied transcription factors NFATc2, c-Fos, c-Jun, and NF-κBp65 have indeed an activating effect on the frequency of IL-2-expressing cells within the FoxP3^+^ cell population. The model and corresponding data are shown in Figure 6. Interestingly, the model predicts that the contribution of all four activating transcription factors for IL-2 expression is similar, because the fitted parameter *c*_1_ is comparable between them.

### Manipulation of c-Fos expression confirms the causal relationship between the level of c-Fos and the probability of IL-2 production per cell in the FOXP3^+^ population

**Figure 7:**
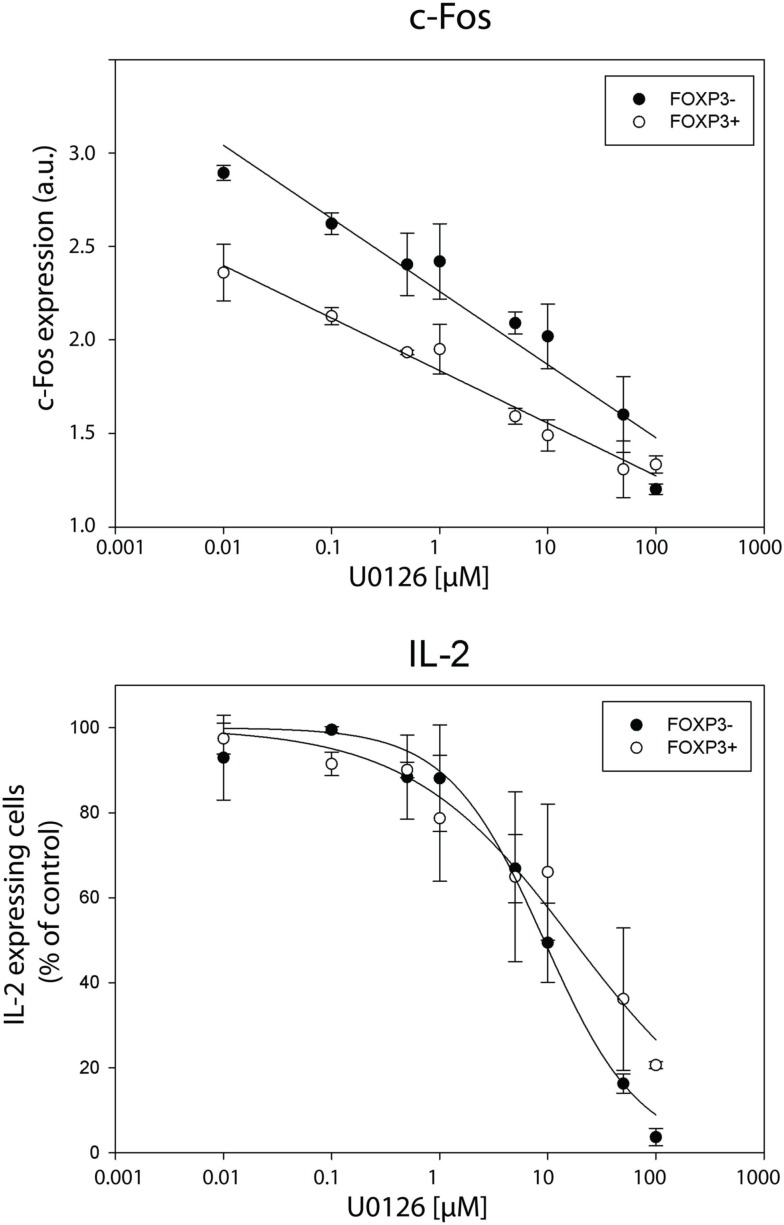
**Inhibition of c-Fos expression diminishes IL-2 expression per cell in both, FOXP3^−^ and FOXP3^+^ subpopulation**. Sorted human CD4+ T cells were pre-incubated with different concentrations of the MEK1/2 inhibitor U0126 for 20 min and subsequently stimulated with PMA/ionomycin for 5 h. Gated memory Th cells (CD45RO^+^ CD45RA^−^) were divided into FOXP3^−^ and FOXP3^+^ cells for analyzing c-Fos and IL-2 expression in these two subpopulations. C-Fos expression and frequencies of IL-2-expressing cells are depicted at different concentrations of U0126. Shown are mean values and the standard deviation of two independent experiments with three replicates each. Regression lines serve for guiding the eye.

In order to confirm that the transcription factor c-Fos is limiting for IL-2 production, we manipulated the physiological c-Fos expression levels using U0126, a specific MEK1/2 small molecular inhibitor. Recently, we confirmed that U0126 does not inhibit the expression of NFATc2, c-Jun, and NF-κBp65 under the conditions used (Bendfeldt et al., 2012). U0126 inhibits *de novo* synthesis of c-Fos after T cell stimulation in a dose-dependent manner (Figure 7, upper part). Simultaneously, the frequency of IL-2 producers declines in both, memory FOXP3^−^ and FOXP3^+^ subpopulation of sorted human CD4^+^ T cells (Figure 7, lower part).

## Discussion

A detailed knowledge of human FOXP3^+^ cell subsets is essential for a better understanding of the regulation of human FOXP3 expression, the study of abnormalities among the FOXP3^+^ subpopulations in autoimmune diseases and allergies, and the selection and purification of the most promising subpopulation(s) of Treg cells for *in vitro* expansion and adoptive transfer (Fujii et al., 2011; Miyara and Sakaguchi, 2011). Recent studies from different groups have demonstrated the heterogeneity of human FOXP3^+^ cells and have begun to define and characterize possible subsets (Baecher-Allan et al., 2001; Ito et al., 2008; Thornton et al., 2010; Akimova et al., 2011; Bianchini et al., 2011; Schuler et al., 2011; Solstad et al., 2011; Duhen et al., 2012).

In our studies, we depicted the IL-2-expressing cell subset within the memory FOXP3^+^ population for further characterization. Our procedure is based on the observation that cytokine production (in particular IL-2 and IFN-γ) and suppression are mutually exclusive functional programs within the FOXP3^+^ T cell population (Gavin et al., 2007; Wan and Flavell, 2007; Williams and Rudensky, 2007). Therefore, we used measurement of anergic behavior as a substitute for suppression assays.

In agreement with others we found that up to 20% of all memory FOXP3^+^ T cells (Miyara et al., 2009; Solstad et al., 2011) and up to 35% of memory FOXP3^low^ T cells express IL-2 after stimulation. The present comparison of IL-2-expressing vs. IL-2-non-expressing cell subsets of the memory FOXP3^+^ population revealed the following main phenotypical and functional differences: IL-2-expressing memory FOXP3^+^ cells have (i) lower levels of FOXP3, (ii) higher levels of NFATc2, c-Fos, c-Jun, and NF-κBp65, (iii) lower levels of CD25 and GITR, and (iv) almost no Helios expression. In contrast to memory Th cells, IL-2 expression is not only limited by endogenous cellular amounts of c-Fos and NFATc2 but in addition by c-Jun and NF-κBp65.

Due to the physiological importance and possible clinical applications of human Treg cells, there have been many studies examining discrimination and characterization of different subsets. So far, the most promising discrimination strategy of human FOXP3^+^ Th cell subsets was proposed by Miyara et al. (2009). The CD45RA^−^FOXP3^low^ subset in particular is still very heterogeneous concerning the phenotypical and functional properties. The memory FOXP3^low^ fraction reveals intermediate characteristics of memory Treg and memory Th cells.

Why is it interesting to subdivide the memory FOXP3^low^ population into further subpopulations? On one hand, this population is very large, comprising approximately 40% of the FOXP3^+^ cells (Miyara et al., 2009). On the other hand this population might contain both specific Th cells and specific Treg cells. The specific CD4^+^ T cells with low FOXP3 expression could be recently activated Th cells (Gavin et al., 2006; Allan et al., 2007; Tran et al., 2007; Wang et al., 2007). The specific memory Treg cells with low FOXP3 expression could be exTreg (former Treg) cells, as suggested by recent publications on murine Th cells (Miyao et al., 2012). The activation-induced up-regulation of FOXP3 in *ex vivo* stimulated human Th cells seems to depend on the strength of stimulation (Gavin et al., 2006; Allan et al., 2007; Wang et al., 2007). ExTreg cells were recently identified in mice (Miyao et al., 2012). These cells represent a minor population of non-regulatory FOXP3^+^ cells losing FOXP3 expression but retaining its memory. ExTreg cells are characterized by a lower expression of certain Treg cell markers, such as FOXP3, CD25, GITR, and Helios, and a higher frequency of IL-2- and IFN-γ-expressing cells than those cells that retain a high FOXP3 expression. Approximately 30% of these exTreg cells regain FOXP3 expression in a TCR-dependent manner. Whether there are human exTregs among the FOXP3^low^ cell population has to be studied.

However, irrespective of the suppressive capacities of “true” FOXP3^+^Treg cells, FOXP3^+^CD25^+^ anergic T cells might play a role in regulating Th cell proliferation, and differentiation anyway by not producing IL-2 on the one side and consuming IL-2 on the other side.

FOXP3 is neither a specific marker for human Treg cells nor is it sufficient to induce stable Treg cells. Ectopic expression of both human FOXP3 isoforms in human CD4^+^ T cells exhibited a profound suppression of IL-2 and IFN-γ production and a partial up-regulation of several Treg cell-associated markers. However, over-expression of FOXP3 did not lead to acquisition of significant suppressor activity *in vitro* (Allan et al., 2005). FOXP3 deletion (Gavin et al., 2007; Williams and Rudensky, 2007) or reduction (Wan and Flavell, 2007) in mice partially recovered the ability of IL-2 and IFN-γ production of Treg cells.

FOXP3 is directly acting on several genes as a transcriptional repressor or activator such as CD25 and CTLA-4, respectively. Our recent data (Bendfeldt et al., 2012) and the present data illustrated in Figures 3 and 5 suggest that the studied transcription factors NFATc2, c-Fos, c-Jun, and NF-κBp65 are downregulated in the FOXP3^+^ population. However, so far it is not known whether these are direct or indirect effects of FOXP3. Interestingly, within the memory FOXP3^−^ population, only the endogenous level of c-Fos and, to a lesser extent, NFATc2 are limiting for IL-2 expression (Bendfeldt et al., 2012). The transcription factors c-Jun and NF-κBp65 occur in such physiological ranges that they do not reduce the probability of IL-2 expression in memory Th cells. This is in contrast to the memory FOXP3^+^ cells described here. In these cells, not only are c-Fos and NFATc2 limiting for IL-2 expression but in addition c-Jun and NF-κBp65. We hypothesize that the concentration of c-Jun and NF-κBp65 shifted from saturation for IL-2 expression in memory FOXP3^−^ cells into a region of limiting concentrations in memory FOXP3^+^ cells. All our studies do not rule out the importance of other transcription factors in the regulation of IL-2 expression such as c-Rel or NFATc1 (Serfling et al., 1989; Randak et al., 1990; Briegel et al., 1991; Hentsch et al., 1992; Kontgen et al., 1995; Rao et al., 2003).

Computational models of transcriptional regulation combined with comprehensive experimental data are helpful in understanding the underlying molecular mechanisms of gene expression. In the present report, we modeled the relation between transcription factor concentration and the fraction of IL-2-expressing cells based on a simple activation and inhibition form. Even with this basic approach we were able to describe the experimental data very well. More importantly, the estimated parameters are in a similar range for each transcription factor, suggesting that their regulatory strength is similar.

Altogether, our results indicate that human IL-2-expressing compared to IL-2-non-expressing FOXP3^+^ cells exhibit a lower level of phenotypical properties of Treg cells, a lower concentration of FOXP3, and higher concentrations of NFATc2, c-Fos, c-Jun, and NF-κBp65. We suggest that these two FOXP3+ cell subsets behave also differently *in vitro* and *in vivo*. Therefore, it is important to further characterize these two subpopulations concerning their proliferative behavior and suppressive capacity as well as in the context of autoimmune diseases (Miyara and Sakaguchi, 2011), if it is possible to separate distinct phenotypic subpopulations within the FOXP3^low^ cell population in sufficient amounts.

## Authors Contribution

Hanna Bendfeldt, Manuela Benary, and Tobias Scheel conducted the experiments and analyzed the data; Hanspeter Herzel and Ria Baumgrass supervised the project; Andreas Radbruch and Kerstin Steinbrink provided essential advice; Ria Baumgrass wrote the paper. All authors read and commented on the draft versions of the manuscript.

## Conflict of Interest Statement

The authors declare that the research was conducted in the absence of any commercial or financial relationships that could be construed as a potential conflict of interest.
